# Buthionine sulfoximine sensitizes antihormone-resistant human breast cancer cells to estrogen-induced apoptosis

**DOI:** 10.1186/bcr2208

**Published:** 2008-12-05

**Authors:** Joan S Lewis-Wambi, Helen R Kim, Chris Wambi, Roshani Patel, Jennifer R Pyle, Andres J Klein-Szanto, V Craig Jordan

**Affiliations:** 1 Department of Medical Sciences, Fox Chase Cancer Center, 333 Cottman Avenue, Philadelphia, PA, 19111, USA; 2 Department of Surgical Oncology, Fox Chase Cancer Center, 333 Cottman Avenue, Philadelphia, PA, 19111, USA; 3 Department of Radiation Oncology, University of Pennsylvania, 195 John Morgan Building, 3620 Hamilton Walk, Philadelphia, PA 19104, USA; 4 Department of Pathology, Fox Chase Cancer Center, 333 Cottman Avenue, Philadelphia, PA, 19111, USA

## Abstract

**Introduction:**

Estrogen deprivation using aromatase inhibitors is one of the standard treatments for postmenopausal women with estrogen receptor (ER)-positive breast cancer. However, one of the consequences of prolonged estrogen suppression is acquired drug resistance. Our group is interested in studying antihormone resistance and has previously reported the development of an estrogen deprived human breast cancer cell line, MCF-7:5C, which undergoes apoptosis in the presence of estradiol. In contrast, another estrogen deprived cell line, MCF-7:2A, appears to have elevated levels of glutathione (GSH) and is resistant to estradiol-induced apoptosis. In the present study, we evaluated whether buthionine sulfoximine (BSO), a potent inhibitor of glutathione (GSH) synthesis, is capable of sensitizing antihormone resistant MCF-7:2A cells to estradiol-induced apoptosis.

**Methods:**

Estrogen deprived MCF-7:2A cells were treated with 1 nM 17β-estradiol (E_2_), 100 μM BSO, or 1 nM E_2 _+ 100 μM BSO combination *in vitro*, and the effects of these agents on cell growth and apoptosis were evaluated by DNA quantitation assay and annexin V and terminal deoxynucleotidyl transferase dUTP nick end-labeling (TUNEL) staining. The in vitro results of the MCF-7:2A cell line were further confirmed *in vivo *in a mouse xenograft model.

**Results:**

Exposure of MCF-7:2A cells to 1 nM E_2 _plus 100 μM BSO combination for 48 to 96 h produced a sevenfold increase in apoptosis whereas the individual treatments had no significant effect on growth. Induction of apoptosis by the combination treatment of E_2 _plus BSO was evidenced by changes in Bcl-2 and Bax expression. The combination treatment also markedly increased phosphorylated c-Jun N-terminal kinase (JNK) levels in MCF-7:2A cells and blockade of the JNK pathway attenuated the apoptotic effect of E_2 _plus BSO. Our *in vitro *findings corroborated *in vivo *data from a mouse xenograft model in which daily administration of BSO either as a single agent or in combination with E_2 _significantly reduced tumor growth of MCF-7:2A cells.

**Conclusions:**

Our data indicates that GSH participates in retarding apoptosis in antihormone-resistant human breast cancer cells and that depletion of this molecule by BSO may be critical in predisposing resistant cells to E_2_-induced apoptotic cell death. We suggest that these data may form the basis of improving therapeutic strategies for the treatment of antihormone resistant ER-positive breast cancer.

## Introduction

Currently, estrogen deprivation using aromatase inhibitors is one of the standard treatments for postmenopausal women with estrogen receptor (ER)-positive breast cancer [1]. Unfortunately, a major clinical problem with the use of prolonged estrogen deprivation is the development of drug resistance (that is, hormone-independent growth) [2,3]. Our laboratory as well as other investigators, have instigated a major effort in studying antihormone resistance in breast cancer and have developed model systems of estrogen deprivation that are sensitive [4-6] or resistant to the apoptotic actions of estrogen [7]. In particular, we have previously reported the development of an estrogen deprived breast cancer cell line, MCF-7:5C, which undergoes estradiol-induced apoptosis after 2 days of treatment via the mitochondrial pathway [8]. In contrast, we have another estrogen deprived breast cancer cell line, MCF-7:2A, which appears to be resistant to estradiol-induced apoptosis [7]. We are studying resistance to estrogen induced apoptosis because clinical experience shows us that only 30% of patients respond to estrogen induced apoptosis once exhaustive antihormonal therapy occurs [9]. An important goal would be to see whether the apoptotic effect of estrogen can be enhanced in antihormone resistant cells. This new, targeted approach to the treatment of metastatic breast cancer could open the door to novel approaches to treatment with drug combinations.

L-Buthionine sulfoximine (BSO) is a specific γ-glutamylcysteine synthetase inhibitor that blocks the rate-limiting step of glutathionine (GSH) biosynthesis and in doing so depletes the intracellular GSH pool in both cultured cells and in whole animals [10]. GSH is a water-soluble tripeptide composed of glutamine, cysteine, and glycine. Reduced glutathione is the most abundant intracellular small molecule thiol present in mammalian cells and it serves as a potent intracellular antioxidant protecting cells from toxins such free radicals [11,12]. Changes in GSH homeostasis have been implicated in the etiology and progression of a variety of human diseases, including breast cancer [13]. In particular, studies have shown that elevated levels of GSH prevent apoptotic cell death whereas depletion of GSH facilitates apoptosis [10,14]. BSO depletes cellular GSH [10] and sensitizes tumor cells to apoptosis induced by standard chemotherapeutic agents [15,16].

Apoptosis (programmed cell death) is required for normal development and tissue homeostasis in multicellular organisms. Deregulation of apoptosis is fundamental to many diseases, such as cancer, stroke, heart disease, neurodegenerative disorders, and autoimmune disorders [17]. There are two main pathways for apoptosis, namely the extrinsic receptor mediated pathway and the intrinsic mitochondria-mediated pathway [18,19]. Components of the extrinsic pathway include the death receptors FasR/FasL, DR4/DR5, and tumor necrosis factor (TNF) [20], whereas the intrinsic pathway centers on the Bcl-2 family of proteins which comprises both proapoptotic proteins, such as Bax, Bak, and Bid and antiapoptotic proteins, such as Bcl-2 and Bcl-xL [18,19]. The Bcl-2 family proteins regulate apoptosis by altering mitochondrial membrane permeabilization which leads to the release of apoptogenic factors such as cytochrome *c*, procaspases, and apoptosis inducing factor (AIF). In particular, Bcl-2 and Bcl-xL inhibit apoptosis by maintaining mitochondrial membrane integrity whereas Bax and Bak facilitate apoptosis by initiating the loss of outer mitochondrial integrity [21]. Apart from its action on the mitochondria, there is also evidence that Bcl-2 possesses antioxidant property. Bcl-2 overexpression increases cellular GSH level which is associated with increased resistance to chemotherapy-induced apoptosis [22,23] whereas GSH depletion restores apoptosis in Bcl-2 expressing cells [16].

Based on microarray studies we found that the antihormone resistant MCF-7:2A cells express markedly elevated levels of glutathione synthetase (GS) and glutathione peroxidase 2 (GPx2); two enzymes that are involved in glutathione synthesis, which suggests that resistance to estrogen-induced apoptosis might be due to elevated levels of GSH present in the cells. If MCF-7:2A cells do indeed possess high levels of GSH, then it is possible that the use of BSO – as a single agent – might be able to sensitize these cells to estrogen-induced apoptosis. As mentioned before, there is current clinical interest in using low dose estradiol therapy to treat antihormone resistant breast cancer [24] however only a minimal 30% of patients respond to this therapeutic strategy. A combination of BSO and estradiol could possibly be used to improve the efficacy of estradiol as an apoptotic agent if glutathione depletion is fundamental to tumor cell survival. We have addressed the hypothesis that by altering glutathione levels we may be able to enhance apoptosis to estrogen and have employed BSO as our agent of choice because of earlier work clinically, which may provide a foundation for subsequent clinical trials.

In the present study, we show that depletion of cellular GSH by BSO sensitizes antihormone-resistant MCF-7:2A cells to estradiol-induced apoptosis that is mediated, in part, by the mitochondrial pathway and also activation of the c-Jun N-terminal kinase (JNK) signaling pathway. We further show that BSO, either alone or in combination with estradiol, causes tumor regression of MCF-7:2A cells *in vivo*.

## Materials and methods

### Cell lines and reagents

The MCF-7 human breast cancer cell line was obtained from Dr Dean Edwards (University of Texas, San Antonio, TX, USA) and was maintained in phenol red RPMI 1640 medium supplemented with 10% fetal bovine serum (FBS), 2 mM glutamine, 100 U/mL penicillin, 100 μg/mL streptomycin, 1 × non-essential amino acids and bovine insulin at 6 ng/mL. The clonal cell line, MCF-7:2A, was derived by growing MCF-7 cells in estrogen-free media for more than 1 year, followed by two rounds of limiting dilution cloning [7]. These cells were grown in phenol red-free RPMI 1640 medium supplemented with 10% 4 × dextran-coated, charcoal-treated FBS (SFS). All reagents for cell culture were obtained from Invitrogen (Life Technologies, Carlsbad, CA, USA). DL-Buthionine sulfoximine (BSO) and 17β-estradiol (E_2_) were from Sigma (St Louis, MO, USA), rhodamine 123 (Rh123) was from Invitrogen (Life Technoligies, Carlsbad, CA, USA). LY294002 and SP600125 were from EMD (Gibbstown, NJ, USA)

### Western blot analysis

The antibodies used for western blotting included those against stress-activated protein kinase (SAPK)/JNK, phospho-SAPK/JNK (Thr183/Tyr185), caspase-7, caspase-9, phospho-Bcl-2 (Ser70), and poly(ADP-ribose) polymerase (PARP) (Cell Signaling Technology, Danvers, MA, USA), cytochrome *c *and β-actin (Sigma, St Louis, MO, USA), cytochrome oxidase subunit IV (Cox IV; Invitrogen, Carlsbad, CA, USA), Bax, Bcl-2, and Bcl-xL (Santa Cruz Biotechnology, Santa Cruz, CA, USA). Western blotting analysis was performed as previously described [8].

### Cell proliferation assays

Proliferation assay was performed as previously described [8]. Briefly, MCF-7 and MCF-7:2A cells were seeded in estrogen-free RPMI media containing 10% SFS at a density of 2 × 10^4 ^cells per well in 24-well plates. After 24 h, cells were treated with the respective drugs for 2, 5, and 7 days with retreatment on alternate days. The DNA content of the cells was determined as previously described [25] using a Fluorescent DNA Quantitation kit (Bio-Rad, Hercules, CA, USA). For each analysis, six replicate wells were used, and at least three independent experiments were performed.

Cell proliferation was also determined by cell counting using a hemocytometer. MCF-7 and MCF-7:2A cells were seeded at a density of 0.5 × 10^6 ^cells in 100 mm dishes and after 24 h cells were treated with 1 nM E_2_, 100 μM BSO, or 1 nM E_2 _plus 100 μM BSO for 7 days with re-treatment on alternate days. For each analysis, three replicate dishes were used, and at least three independent experiments were performed.

### Detection of apoptosis by annexin V staining

The annexin V-fluorescein isothiocyanate (FITC) labeled Apoptosis Detection Kit I (BD Biosciences, San Jose, CA, USA) was used to detect and quantify apoptosis by flow cytometry, according to the manufacturer's instructions.

### Terminal deoxynucleotidyl transferase-mediated dUTP nick end-labeling (TUNEL) staining for apoptosis

Apoptosis was also determined by the TUNEL assay using an *in situ *cell death detection kit conjugated with horse-radish peroxidase (POD) (Roche Applied Science, Indianapolis, IN, USA), according to the manufacturer's instructions. Briefly, fixed cells were washed, permeabilized, and then incubated with 50 μL of terminal deoxynucleotidyl transferase end-labeling cocktail for 60 min at 37°C in a humidified atmosphere in the dark. For signal conversion, slides were incubated with 50 μL of converter-POD (anti-fluorescein antibody conjugated with horseradish peroxidase) for 30 min at 37°C, rinsed with PBS, and then incubated with 50 μL of 3,3'-diaminobenzidine (DAB) substrate solution for 10 min at 25°C. The slides were then rinsed with phosphate-buffered saline (PBS), mounted under glass coverslips, and analyzed under a light microscope using an inverted Nikon TE300 (Nikon, Melville, NY, USA).

### GSH assay

Total cellular GSH was measured using the Total Glutathione Colorimetric microplate assay Kit (Oxford Biomedical Research), according to the manufacturer's protocol. Cells were plated at 0.5 × 10^6^/well of a six-well plate and allowed to recover overnight. After appropriate treatments, cells were washed in PBS and then lysed in 100 to 150 μl of buffer (100 mM NaPO_4_, 1 mM ethylenediaminetetraacetic acid (EDTA), pH 7.5) containing 0.1% Triton X-100 and frozen at -80°C until analysis. To measure total glutathione, proteins were precipitated with sulfosalicylic acid at a final concentration of 1%. Samples were then spun for 10 min in a microcentrifuge to pellet proteins, and supernatant was diluted 1:20 in buffer before being measured. For all measurements, 50-μl triplicates of each sample were used for glutathione determination. The GSH level was obtained by comparing with the GSH standards and represented as nmol/mg of protein.

### Mitochondrial transmembrane potential (ΔΨ_m_) and cytochrome c release

Changes in the mitochondrial membrane potential (ΔΨm) were examined by monitoring the cells after staining with rhodamine 123. Briefly, estradiol plus BSO-treated MCF-7:2A cells were washed twice with PBS and incubated with 1 μg/mL rhodamine 123 at 37°C for 30 min. Cells were then washed twice with PBS, and Rh123 intensity was determined by flow cytometry. Cells with reduced fluorescence were counted as having lost some of their mitochondrial membrane potential.

For cytochrome *c *release assays, cells were lysed in lysis buffer (10 mmol/L *N*-2-hydroxyethylpiperazine-*N*'-2-ethanesulfonic acid (HEPES; pH 7.5), 10 mmol/L KCl, and 1 mmol/L EDTA) with protease inhibitor cocktail (Sigma), frozen and thawed three times, and centrifuged at 2,000 *g *for 5 min. The supernatants were centrifuged at 10,000 *g *for 15 min at 4°C, and the mitochondrial pellets were dissolved in sodium dodecyl sulfate (SDS) sample buffer, subjected to 15% SDS-polyacrylamide gel electrophoresis (SDS-PAGE), and analyzed by immunoblotting with monoclonal antibodies against cytochrome *c *and Cox IV.

### RNA isolation and quantitative real-time polymerase chain reaction (PCR)

Total RNA was isolated using TRI reagent (Invitrogen) according to the manufacturer's protocol. RNA (2 μg) was reverse transcribed to cDNA using the SuperScript II RNase H-reverse transcriptase system (Invitrogen, Carlsbad, CA, USA). Aliquots of the cDNA were combined with the SYBR green kit and primers, and assayed in triplicate by real-time quantitative PCR using a GeneAmp^® ^5700 Sequence detection system (Applied Biosystems Inc, Foster City, CA, USA). Quantitation was performed using the comparative threshold cycle (Ct) method with 18S rRNA as the normalization gene, as previously described [8]. GS and GPx2 primers were designed using Primer Express™ software following the manufacturer's guidelines. Primers were synthesized by Applied Biosystems. Quantitative PCR was performed using the following conditions: 40 cycles; denaturation at 95 C for 15 s, annealing at 63 C for 1 min, and polymerization at 72 C for 1 min. Primer sequences were: GS forward: CACCAGCT GGGGAAGCATCT; reverse: GGTGAGGGGAAGAGCGT GAA, GPx2 forward: TTG ATT AAG GCT TTC TTT GGT AGG; reverse: TTT CAA TAA ATC AGG TCC CAG G.

### Small interfering RNA (siRNA) transfection

Bcl-2-specific siRNA was chemically synthesized by Dharmacon Inc (Chicago, IL, USA). A non-targeting siRNA duplex was used as negative control. For transfection, MCF-7:2A cells were seeded in complete medium without antibiotics the day before the experiment in 12-well plates at a density of 70,000 cells per well. After 24 h, cells were transfected with 100 nM of Bcl-2 siRNA or control siRNA, using DharmaFect 1 transfection reagent (Dharmacon Inc, Chicago, IL, USA), according to the manufacturer's protocol. The cells were harvested 48 h post transfection and analyzed by western blot. Transfected cells were also treated with estradiol for an additional 72 h and apoptotic cells were measured using annexin V staining.

### Inhibition of MCF-7:2A cell tumorigenesis by BSO in nude mice

Female CrTac:NCr-Foxn1nu athymic mice (4 to 5 weeks old) were purchased from Taconic (Germantown, NY, USA). Animal experiments were conducted at the Fox Chase Cancer Center (Philadelphia, PA, USA). The research protocol was approved, and mice were maintained in accordance with institutional guidelines of the Fox Chase Cancer Center Animal Care and Use Committee. Mice were acclimatized to the animal facility for 1 week before they received injections of MCF-7:2A human breast cancer cells: 2 × 10^7 ^cells were resuspended in 100 μL PBS (Collaborative Biomedical Products, Bedford, MA, USA) and were bilaterally injected into the mammary fat pads of 20 ovariectomized mice. Tumors were allowed to develop for 20 days until they reached a mean cross-sectional area of 0.32 cm^2^, when treatment was initiated with placebo (saline), E_2 _(0.3 cm capsule), BSO (4 mmol/kg weight), or BSO (4 mmol/kg weight) plus E_2 _(0.3 cm capsule) for an additional 7 days. For the estradiol treatment, 0.3 cm silastic estradiol capsules (Baxter HealthCare, Mundeleine, IL, USA) were implanted subcutaneously in the mice. These capsules produced a mean serum estradiol level of 83.8 pg/mL [26], to achieve postmenopausal serum levels of estradiol. BSO was dissolved in saline and was administered intraperitoneally daily for 7 days. The cross-sectional tumor area was calculated by multiplying the length (*l*) by the width (*w*) by π and dividing the product by 4 (*lwπ*/4). Animals were given food and water *ad libitum*. Mice from each group (n = 5) were killed at the conclusion of the experiment and immunohistochemical analysis was performed.

### Tissue preparation and immunohistochemistry

Tumors from mice treated with placebo, E_2_, BSO, or BSO plus E_2 _were excised and fixed in 10% formalin, embedded in paraffin wax blocks and sectioned. Subsequently, sections of the tumor blocks were stained with hematoxylin and eosin (H&E), Ki67, or PARP antibody (1:500 dilution, Santa Cruz Biotechnology, Santa Cruz, CA, USA) by the pathology core facility at Fox Chase Cancer Center.

### Statistical analysis

Statistical analysis was performed using the Student t test, and a p value of < 0.05 was considered significant. Data are expressed as the mean ± standard error of the mean (SEM). The mean value was obtained from at least three independent experiments.

## Results

### Estrogen deprivation increases glutathione levels in MCF-7:2A breast cancer cells

**Figure 1:**
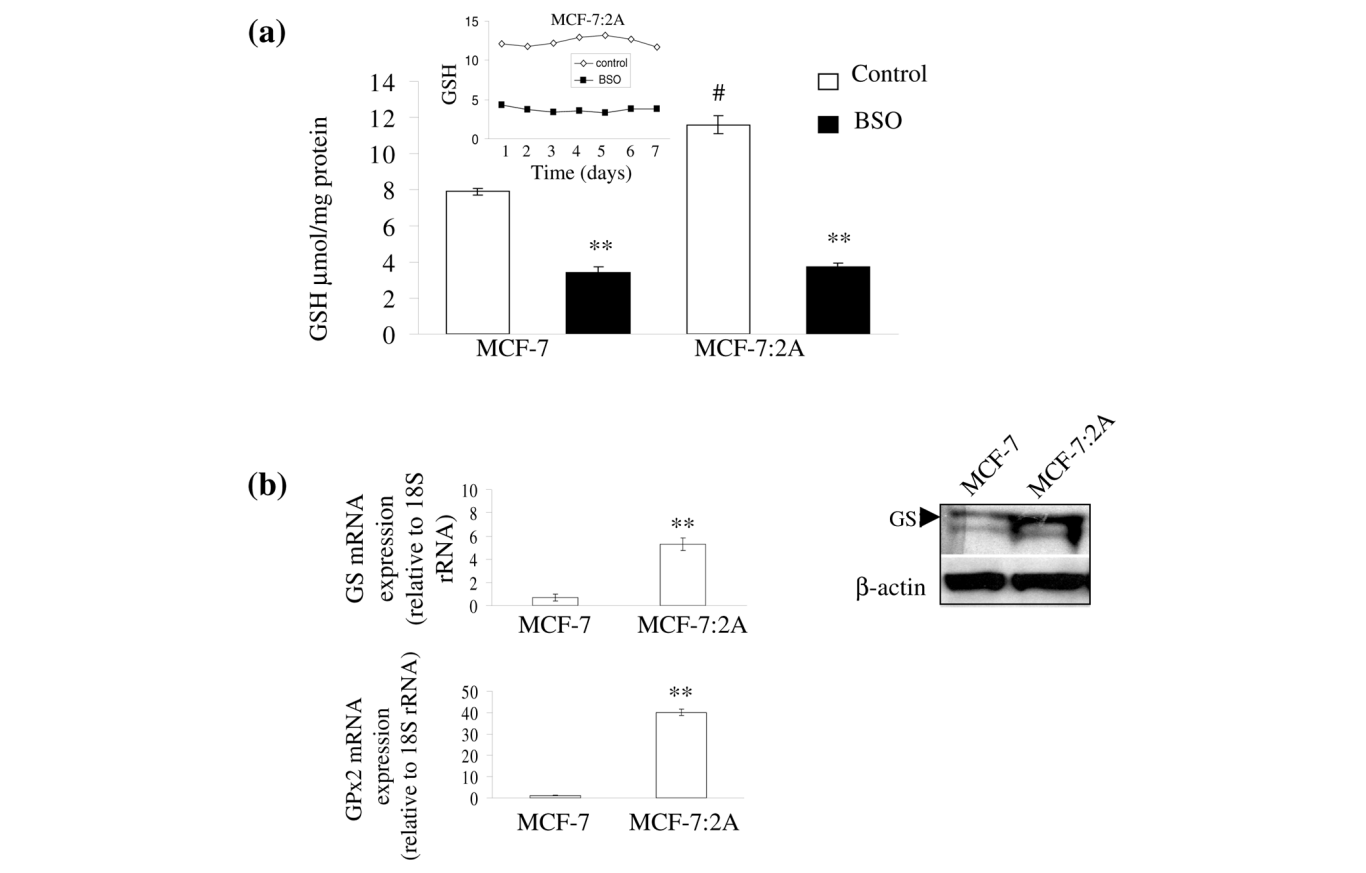
Intracellular glutathione (GSH) levels in wild-type MCF-7 cells and antihormone-resistant MCF-7:2A breast cancer cells. (a) MCF-7 and MCF-7:2A cells were seeded at 2 × 10^6 ^cells per 100 mm culture plates in phenol red RPMI media containing 10% fetal bovine serum (FBS) and phenol red-free RPMI media containing 10% 4× dextran coated charcoal-treated FBS (SFS), respectively, and after 24 h were treated with nothing (control) (white columns) or 100 μM buthionine sulfoximine (BSO) (black columns) for 24 h. Total cellular glutathione was measured using a Glutathione Colorimetric microplate assay kit, as described in Materials and methods. Columns, mean from three separate experiments; bars, ± standard error of the mean (SEM). **, p < 0.001 compared with control cells; ^#^, p < 0.05 compared with MCF-7 control cells. Insert graph shows glutathione levels in MCF-7:2A cells over a 7-day period. (b) Quantitative real-time polymerase chain reaction (PCR) of glutathione sythetase (GS) (top left) and glutathione peroxidase 2 (GPx2) (bottom left) mRNA expression in MCF-7 and MCF-7:2A cells. **, p < 0.001 compared with MCF-7 control cells. Western blot analysis of GS protein expression in MCF-7:2A cells is also shown (top right).

Elevated glutathione levels and the activity of its related enzymes have been characterized as one of the factors which could render breast cancer cells resistant to apoptosis. We have previously shown that MCF-7:2A breast cancer cells are resistant to estrogen-induced apoptosis [7], therefore we measured glutathione levels in these cells along with parental MCF-7 cells. Figure 1a showed that glutathione levels were significantly higher in MCF-7:2A cells (11.9 μM/mg protein) compared to MCF-7 cells (7.8 μM/mg protein) and treatment with BSO (100 μM), an inhibitor of glutathione synthesis, for 24 h depleted glutathione content by approximately 55% and 68% in MCF-7 and MCF-7:2A cells, respectively. It is worth noting that glutathione levels were consistently elevated in MCF-7:2A cells up to 7 days and the inhibitory effect of BSO persisted throughout that incubation period (Figure 1a, insert).

We next examined whether the expression of glutathione-related enzymes was altered in these cells. Using quantitative real-time PCR, we found a 6-fold increase in glutathione synthetase (GS) expression and a 40-fold increase in glutathione peroxidase 2 (GPx2) expressions in MCF-7:2A cells compared to parental MCF-7 cells (Figure 1b). Western blot analysis also showed a marked increase in GS protein level in MCF-7:2A cells compared to parental MCF-7 cells (Figure 1b, right panel).

### BSO enhances the apoptotic effect of E_2 _in MCF-7:2A cells

**Figure 2:**
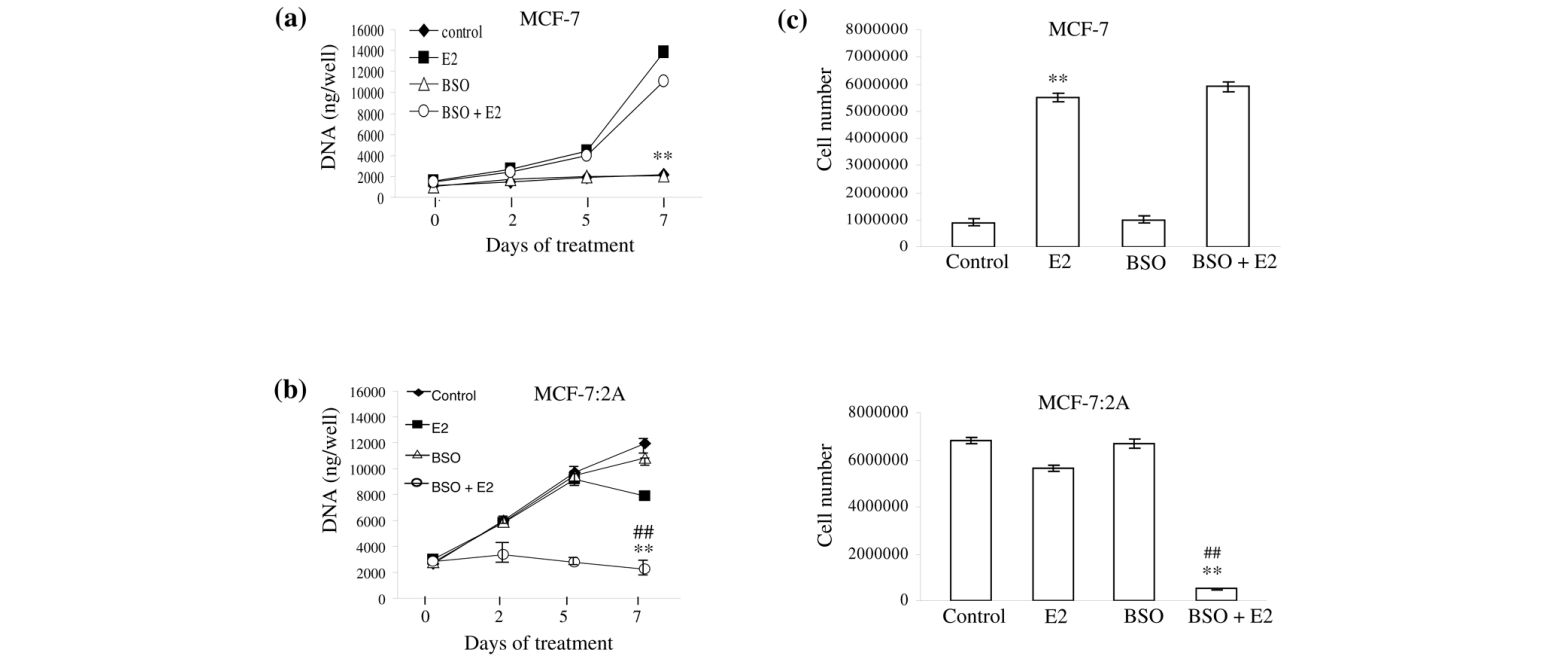
Effect of buthionine sulfoximine (BSO) plus estradiol on the growth of wild-type MCF-7 cells and antihormone-resistant MCF-7:2A cells. (a) MCF-7 cells were grown in estrogen-free media for 3 days prior to the start of the growth assay. On the day of the experiment, 30,000 cells were seeded in 24-well plates and after 24 h were treated with < 0.1% ethanol vehicle (control), 1 nM 17β-estradiol (E_2_), 100 μM BSO, or 100 μM BSO plus 1 nM E_2 _for 7 days. At the indicated time points, cells were harvested and total DNA (ng/well) was quantitated as described in Materials and methods. The data represent the mean of three independent experiments; bars, ± standard error of the mean (SEM). **, p < 0.001 compared with control cells. (b) MCF-7:2A cells were seeded at the same density as MCF-7 cells and were treated similarly. The data represent the mean of three independent experiments; bars, ± SEM. **, p < 0.001 compared with control cells; ^##^, p < 0.001 compared with estradiol-treated cells. (c) The effect of BSO plus estradiol on cell proliferation was also determined by cell counting using a hemocytometer. For experiment, 0.5 × 10^6 ^MCF-7 (top) and MCF-7:2A (bottom) cells were seeded in 15-cm dishes and after 24 h were treated with 1 nM estradiol, 100 μM BSO, or E_2 _plus BSO combination for 7 days. Data shown represents the mean of three independent experiments; bars, ± SEM. **, p < 0.001 compared with control cells; ^##^, p < 0.001 compared with estradiol-treated cells.

We next examined whether depletion of glutathione levels by BSO sensitizes MCF-7:2A cells to estrogen-induced apoptosis. For proliferation assays, MCF-7 and MCF-7:2A cells were seeded in estrogen-free media, and after 24 h, were treated with 100 μM BSO, 1 nM E_2_, or 100 μM BSO plus 1 nM estradiol for 2, 5, and 7 days. Figure 2a shows that the growth of parental MCF-7 cells was stimulated sevenfold over the control cells by 1 nM estradiol during the course of the 7-day assay and that treatment with BSO, either alone or in combination with estradiol, did not significantly alter the growth of these cells. In contrast, MCF-7:2A cells treated with the combination of 100 μM BSO and 1 nM estradiol showed a significant time-dependent decrease in cell growth relative to cells treated with either estradiol or BSO alone. The growth inhibitory effect of BSO and estradiol was observed as early as 48 h after treatment and persisted over the time course of the experiment with maximum cell death at the 7-day time point. The combination of estradiol plus BSO also significantly reduced the proliferation of MCF-7:2A cells (Fig. 2c, bottom) but it did not affect the growth of wild type MCF-7 cells (Figure 2c, top). Furthermore, we found that treatment with the antiestrogen 4-hydroxytamoxifen (4-OHT) almost completely reversed the growth inhibitory effect of estradiol and BSO in MCF-7:2A cells (see Additional data file 1) which suggests the involvement of the ER in this process.

**Figure 3:**
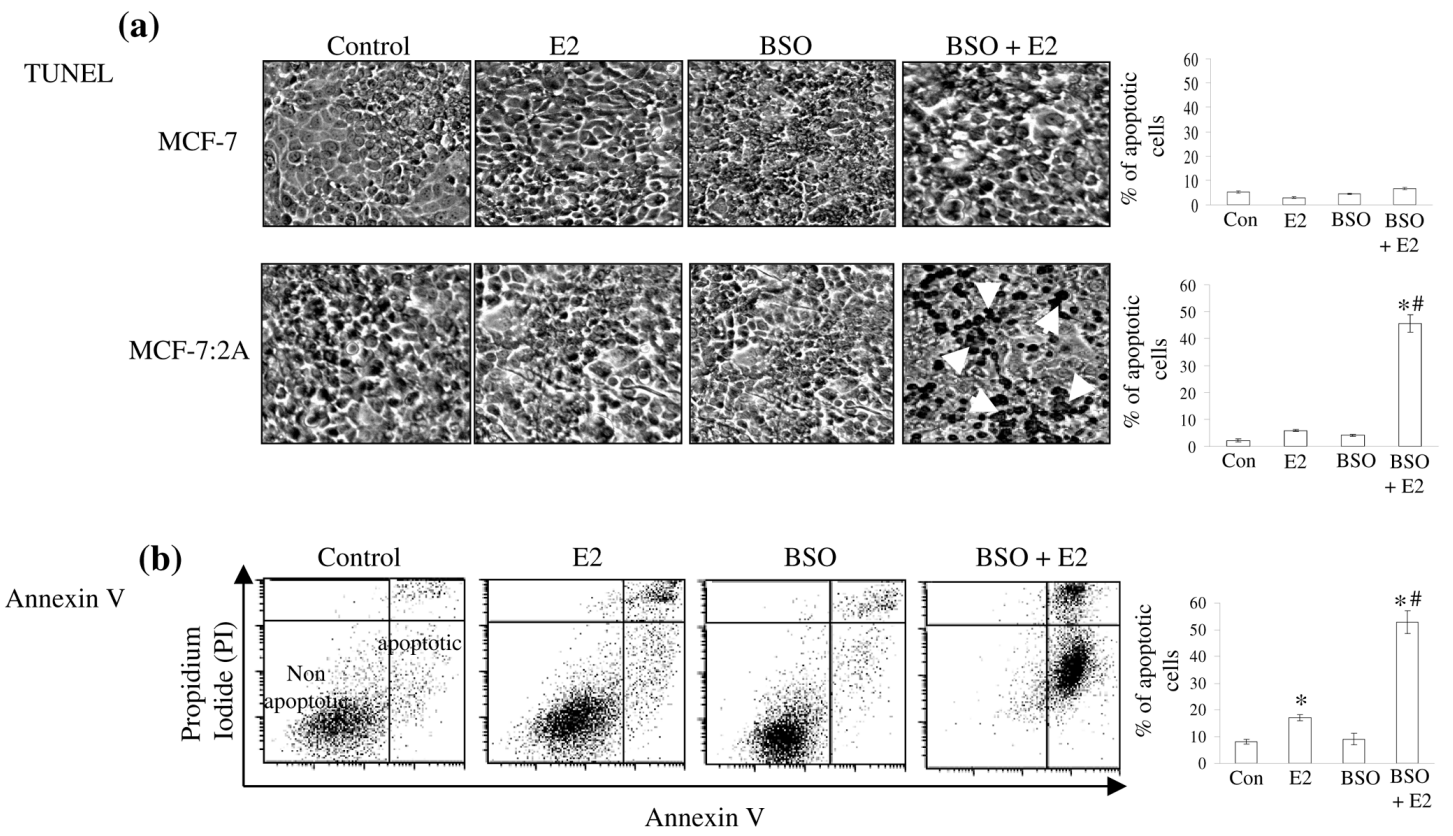
Buthionine sulfoximine (BSO) plus estradiol induce apoptosis in MCF-7:2A cells. (a) Terminal deoxynucleotidyl transferase-mediated dUTP nick end-labeling (TUNEL) staining for apoptosis in MCF-7:2A cells following BSO plus 17β-estradiol (E_2_) treatment for 96 h were performed as described in Materials and methods. Slides were photographed through a brightfield microscope under 100 × magnification. TUNEL-positive cells were stained black (white arrows). Columns (right), mean percentage of apoptotic cells (annexin V-positive cells) from three independent experiments performed in triplicate; bars, ± standard error of the mean (SEM). *, p < 0.001 compared with control cells; ^#^, p < 0.001 compared with estradiol-treated cells. (b) Annexin V staining for apoptosis. Cells were seeded in 100 mm plates at a density of 1 × 10^6 ^per plate and after 24 h were treated with ethanol vehicle (control), 1 nM E_2_, or BSO plus E_2 _for 72 h and then stained with fluorescein isothiocyanate (FITC)-annexin V and propidium iodide (PI) and analyzed by flow cytometry. PI was used as a cell viability marker. Representative cytograms are shown for each group. Quantitation of apoptosis (percentage of control) in the different treatment groups is shown on the right. bars, ± SEM. *, p < 0.05 compared with control cells; ^#^, p < 0.01 compared with estradiol-treated cells.

Based on the above finding, we next determined whether MCF-7:2A cells underwent apoptotic cell death upon BSO and estradiol treatment. We performed a TUNEL assay, which detects the fragmentation of DNA, which is characteristic of cells undergoing apoptotic cell death. As shown in Figure 3a, the percentage of TUNEL-positive cells significantly increased with the combination of BSO and estradiol but not with estradiol or BSO alone. After treatment with BSO and estradiol (96 h), as many as 53% of cells displayed TUNEL-positive staining, whereas, only 1% of the control cells and 5% of the estradiol-treated cells were TUNEL-positive. BSO-treated cells looked similar to control cells. As expected, parental MCF-7 cells showed very little TUNEL-positive staining in the presence of estradiol alone or BSO plus estradiol combined (Figure 2b, top panel), thus indicating a lack of apoptosis in these cells.

To further substantiate the apoptotic effect of BSO and estradiol in MCF-7:2A cells, annexin V-PI immunostaining was performed by flow cytometry. Figure 3b shows that in the BSO plus estradiol-treated group, approximately 55.6% of cells stained positive for annexin V whereas in the control group and estradiol-treated group, approximately 7.4% and approximately 15.6%, respectively, of cells stained positive for annexin V. For the BSO-treated group, only 8.7% of cells stained positive for annexin.

### Role of the mitochondrial pathway in BSO plus estradiol-induced apoptosis in MCF-7:2A cells

**Figure 4:**
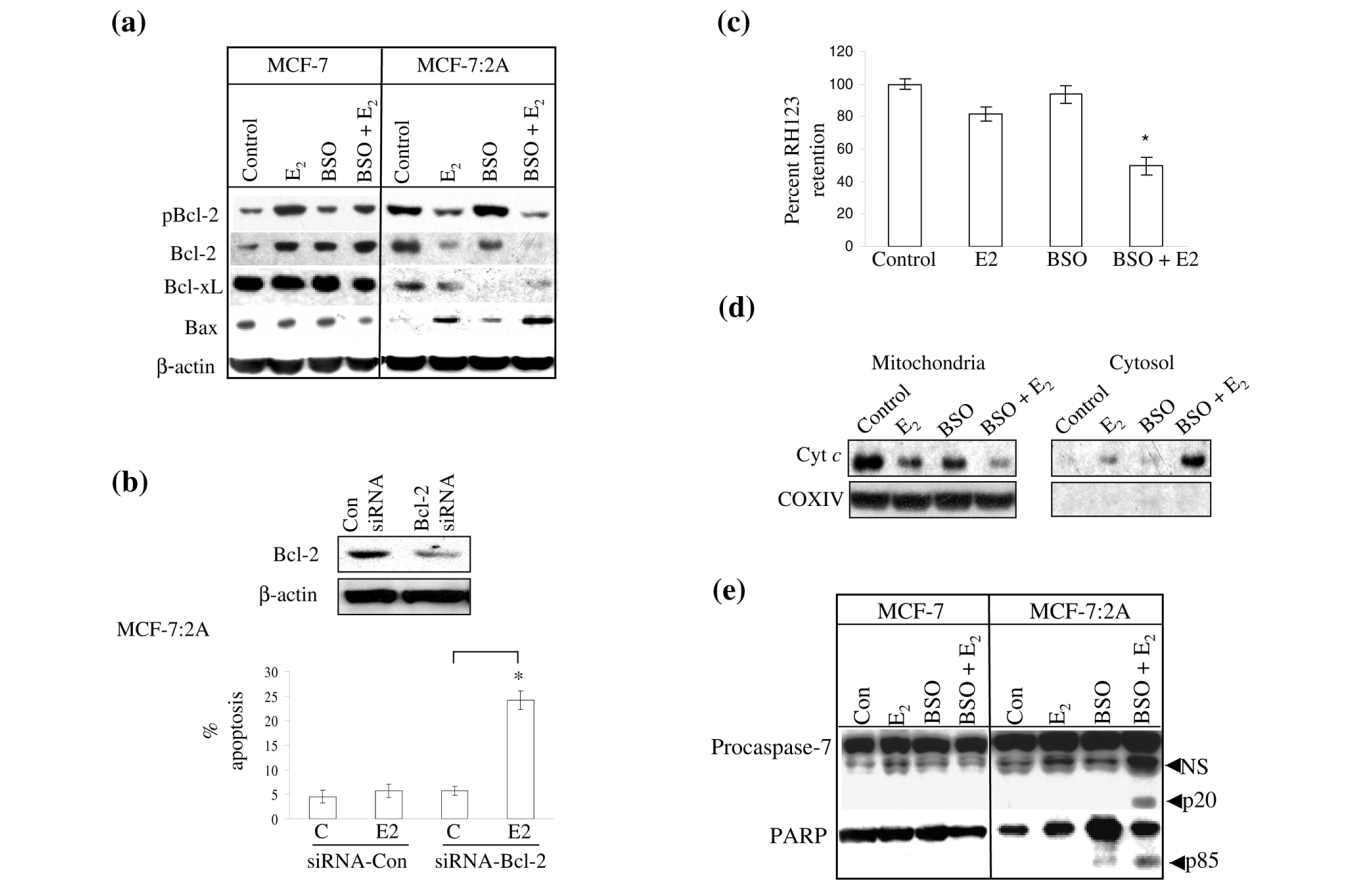
Effect of buthionine sulfoximine (BSO) and 17β-estradiol (E_2_) on Bcl-2 family protein expression and mitochondrial function in MCF-7 and MCF-7:2A cells. (a) Western blot analysis for pBcl-2, Bcl-2, Bcl-x_*L*_, and Bax protein expression in parental MCF-7 cells and MCF-7:2A cells following 48 h of treatment with ethanol vehicle (Control), 1 nM E_2_, 100 μM BSO, or E_2 _+ BSO. Equal loading was confirmed by reprobing with an antibody against β-actin. (b) Small interfering RNA (siRNA) knockdown of Bcl-2 partially sensitizes MCF-7:2A cells to E_2_-induced apoptosis. Cells were transfected with 100 nM siRNA-Bcl-2 or siRNA-Con (control) and expression levels of Bcl-2 was determined by immunoblot analysis (top). Annexin V staining (bottom) showing the effects of siRNA-con and siRNA-Bcl-2 on apoptosis induced by estradiol treatment in MCF-7:2A cells. *, p < 0.001. (c) Loss of mitochondrial potential in MCF-7:2A cells was determined by rhodamine 123 (Rh123) retention assay. The percentage of cells retaining Rh123 in each treatment group was compared with untreated control. (d) Cytochrome *c *release from the mitochondria to the cytosol after treatment with E_2 _alone or BSO and E_2 _for 48 h was determined as described in Materials and methods. Anti-Cox IV antibody was used as a control to demonstrate that mitochondrial protein fractionation was successfully achieved. (e) Cleavage of caspase 7 and poly(ADP-ribose) polymerase (PARP) (72 h) was assessed by western blot using specific antibodies. The upper band of caspase 7 represents the full-length protein and the lower band (p20, arrow) represents the cleaved activated product; NS, nonspecific. Full length PARP is approximately 116 kDa; cleaved (active) PARP is 85 kDa (arrow). The results are representative of three independent experiments.

To examine the role of the mitochondrial pathway in BSO plus estradiol-induced apoptosis, western blot analyses was used to measure Bax, Bcl-2, phosphorylated Bcl-2, and Bcl-xL protein levels in MCF-7:2A cells following treatment with 1 nM estradiol alone, 100 μM BSO, or BSO plus estradiol for 48 h. We found that Bcl-2, phospho-Bcl-2, and Bcl-xL protein levels were almost completely reduced in MCF-7:2A cells treated with BSO plus estradiol compared to control, BSO, or estradiol alone. In addition, a marked increase in Bax expression was also observed in MCF-7:2A cells following BSO plus estradiol combined treatment (Figure 4a). In contrast, similar experiments performed with parental MCF-7 cells showed that BSO plus estradiol slightly increased Bcl-2 and phospho-Bcl-2 protein levels in these cells with a more dramatic effect observed with estradiol alone (Figure 4a). It is worth noting that in MCF-7:2A cells endogenous levels of Bcl-2 and phosphorylated Bcl-2 were markedly elevated compared to parental MCF-7 cells. This finding is consistent with previous reports which show that overexpression of Bcl-2 increases glutathione levels and inhibits mitochondrial dysfunction and cell death elicited by glutathione-depleting reagents [27].

Although estradiol, as an individual treatment, did not significantly induce apoptosis in MCF-7:2A cells, it did decrease Bcl-2 protein level in these cells. We therefore tested whether siRNA knockdown of Bcl-2 expression would sensitize MCF-7:2A cells to estradiol-induced apoptosis. Expression of Bcl-2 following knockdown was analyzed by western blotting. As expected, Bcl-2 protein levels were significantly reduced following transfection of MCF-7:2A cells with Bcl-2 siRNA compared to control siRNA (Figure 4b, top panel). Using annexin V staining, we found that apoptosis was increased by 20% in Bcl-2 siRNA transfected cells compared with cells transfected with the control siRNA (Figure 4b, bottom panel), thus suggesting that suppression of antiapoptotic factors such as Bcl-2 has the ability to partially sensitize hormone-independent MCF-7:2A cells to apoptosis.

We next examined mitochondrial membrane integrity using the Rh123 retention assay. Cells were treated with nothing (control), estradiol, BSO, or BSO plus estradiol for 48 h. Figure 4c shows that BSO plus estradiol treatment reduced Rh123 fluorescence in MCF-7:2A cells by approximately 50% compared to control, whereas, estradiol or BSO, as individual treatments, did not significantly alter Rh123 retention levels in these cells. BSO plus estradiol also enhanced cytochrome *c *release in MCF-7:2A cells. Figure 4d shows that in the control cells, cytochrome *c *was detected primarily in the mitochondria and was undetectable in the cytosol; however, in the presence of BSO plus estradiol (48 h), all of cytochrome *c *was observed in the cytosol. BSO or estradiol, as individual treatments, did not significantly alter mitochondrial release of cytochrome *c*. The translocation of cytochrome *c *from the mitochondria to the cytosol following BSO plus estradiol treatment coincided with cleavage of caspase 7 and PARP (Figure 4e), which is a molecular signature of apoptosis. Cleavage of PARP and caspase 7 was blocked by the pan-caspase inhibitor z-VAD (data not shown).

### The apoptotic effect of BSO and estradiol in MCF-7:2A cells is regulated, in part, by JNK signaling

**Figure 5:**
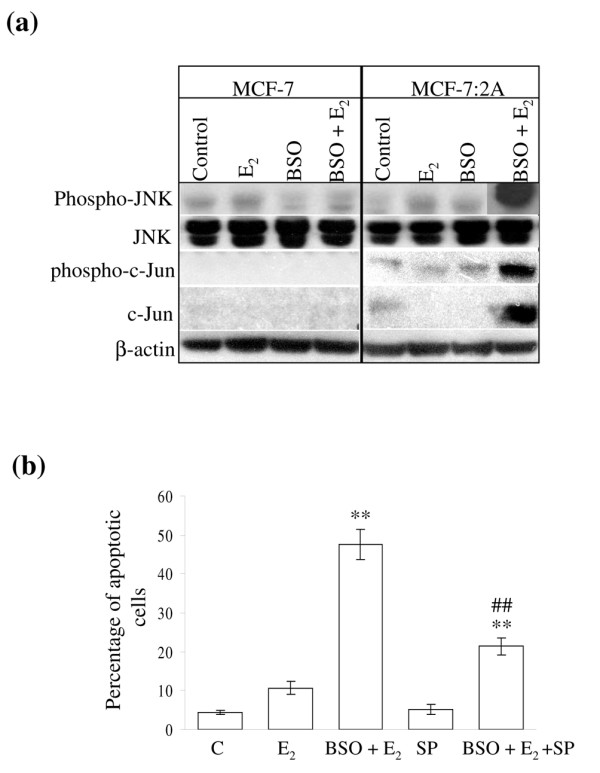
Activation of c-Jun N-terminal kinase (JNK) signaling pathway in MCF-7:2A cells in response to buthionine sulfoximine (BSO) and 17β-estradiol (E_2_) treatment. (a) MCF-7 and MCF-7:2A cells were treated with ethanol vehicle (control), 1 nM E_2 _or 100 μM BSO plus E_2 _for 48 h and protein levels of phosphorylated JNK, JNK, phosphorylated c-Jun, and c-Jun were analyzed by western blotting. β-Actin was used as a control. (b) Inhibition of JNK activation by SP600125 (SP) partially reverses the apoptotic effect of BSO and estradiol in MCF-7:2A cells. Cells were pretreated with 20 μM SP600125 or vehicle for 24 h, then further incubated for 48 h with 1 nM E_2_, E_2 _+ 100 μM BSO, 20 μM SP, or E_2 _+ BSO + SP and apoptosis was determined by annexin V-propidium iodide (PI) staining as described in Materials and methods. Columns, mean percentage of apoptotic cells from three independent experiments performed in triplicate; bars, ± standard error of the mean (SEM). **, p < 0.001 compared with control (C) cells; ^##^, p < 0.01 compared with E_2 _plus BSO-treated cells.

Emerging evidence supports a role for JNK in stress-induced mitochondrial apoptotic pathways in a variety of cell systems [28]. Therefore, we examined the possible involvement of c-Jun/JNK pathway in BSO plus estradiol-induced apoptosis in MCF-7:2A cells. JNK activation was determined by western blot analysis after 48-h treatment of cells with BSO plus estradiol. A profound induction of the p54 and p46 isoforms of phosphorylated JNK as well as a significant increase in phospho-c-Jun and c-Jun were observed in MCF-7:2A cells treated with BSO plus estradiol compared to BSO alone or control (Figure 5a). Interestingly, treatment with estradiol alone also significantly increased phosphorylated JNK in MCF-7:2A cells. We also found that pretreatment of MCF-7:2A cells with the JNK inhibitor, SP600125 (20 μM) markedly reduced the apoptotic effect of BSO plus estradiol in these cells (Figure 5b). Overall, these results suggest a possible involvement of the c-Jun/JNK signaling pathway in BSO plus estradiol-induced apoptosis in MCF-7:2A cells.

### BSO inhibits the growth of MCF-7:2A cells *in vivo*

**Figure 6:**
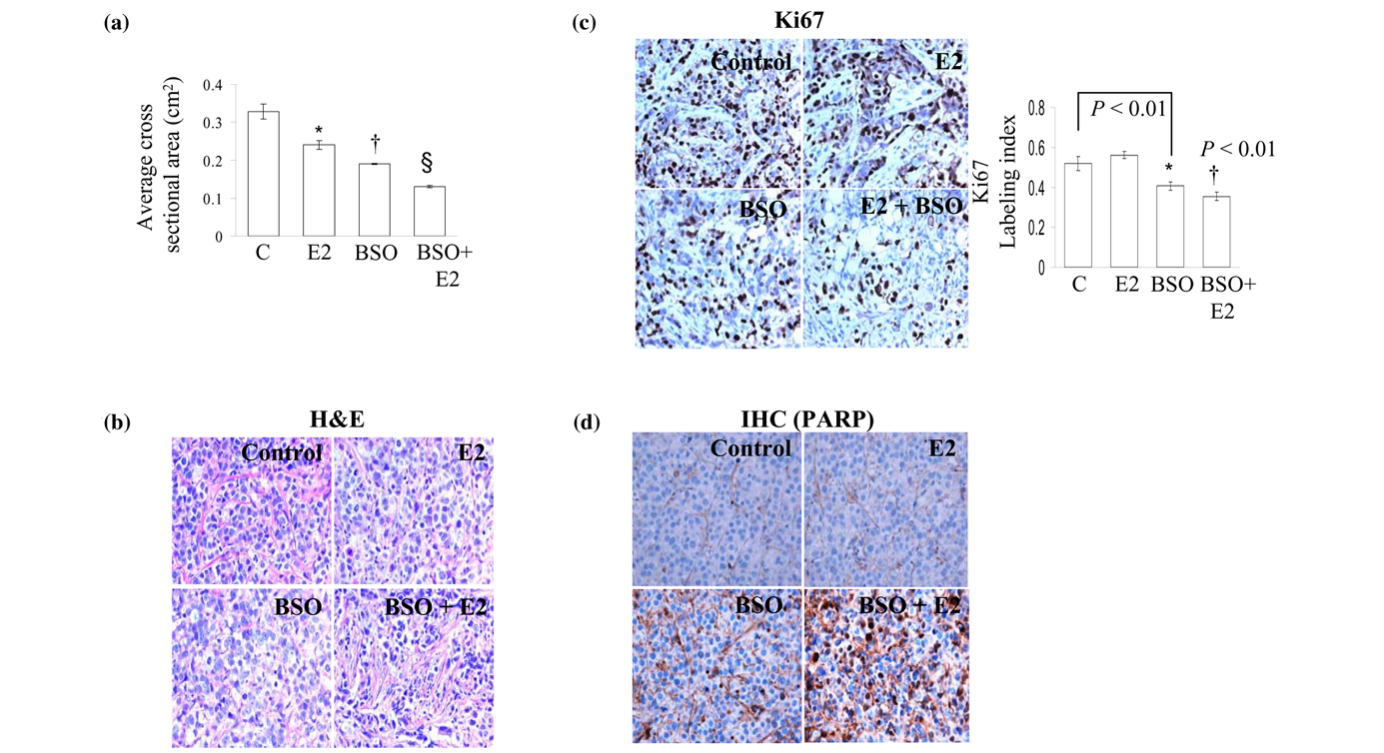
Buthionine sulfoximine (BSO) inhibits the growth of MCF-7:2A tumors in vivo. Athymic nude mice (4 to 5 weeks old, n = 20) were injected with MCF-7:2A breast cancer cells and after 20 days when tumors had reached a mean cross-sectional area of 0.3 cm^2^, animals were randomized into 4 groups and were treated with placebo (saline), 17β-estradiol (E_2_), BSO, or BSO plus E_2 _for 7 days as described in Materials and methods. BSO (4 mmol/kg weight) was diluted in saline and was injected intraperitoneally daily. (a) Tumor size was measured everyday and cross-sectional area was calculated by multiplying the length (*l*) by the width (*w*) by π and dividing the product by 4 (*lwπ*/4). Data is shown as mean ± standard error of the mean (SEM). *, p < 0.05, control group compared with the E_2 _group; †, p < 0.002 control group compared with BSO group; § p < 0.001 control group compared with BSO + E2 group. (b) Microscopy of hematoxylin and eosin (H&E)-stained histological sections of MCF-7:2A tumors treated with placebo, E_2_, BSO, or BSO plus E_2_. (c) Immunohistochemical analysis of the proliferation marker Ki-67 in MCF-7:2A tumors treated with placebo, E_2_, BSO, or BSO plus E_2_. (d) Paraffin-embedded tumor sections of mice treated with E_2_, BSO, or BSO plus E_2 _were immunostained for proteolytically cleaved poly(ADP-ribose) polymerase (PARP), which exists only when cells undergo apoptosis. Three to four tumors per treatment group were analyzed.

To determine whether the effect of BSO plus estradiol was relevant *in vivo*, we used a xenograft model in which MCF-7:2A cells were injected into CrTac:NCr-Foxn1nu athymic mice (n = 20). At 20 days post injection, tumors grew to a mean cross-sectional area of 0.30 cm^2 ^and mice were randomized to four groups; placebo (saline), estradiol, BSO, or the combination of BSO plus estradiol, as described in materials and methods. After 7 days of treatment, tumor growth was reduced by 25% in mice treated with estradiol alone whereas in the BSO and BSO plus estradiol group tumor growth was reduced by 40% and 60%, respectively, compared to the placebo group which showed a 7% increase in growth (Figure 6a). Interestingly, we found that BSO *in vitro *had a relatively small effect on growth, however, *in vivo *its effect was very pronounced, thus suggesting the possibility of altered glutathione metabolism *in vivo*. We performed histology on tumors taken from placebo, estradiol, BSO, or BSO plus estradiol groups at day 27. H&E staining of the BSO plus estradiol-treated tumors revealed less tumor cells and more intercellular matrix, significantly less mitoses, chromatin clumping and dark staining which are associated with apoptosis, and enhanced abnormalities in shape and size, compared to tumors from placebo or BSO or estradiol-treated groups (Figure 6b). We also characterized the proliferative status of these cells by staining tumors for the expression of Ki67, a marker of cell proliferation. We observed a 32% decrease (p < 0.001) in the number of Ki67 stained tumors from the BSO plus estradiol-treated group and a 21% decrease in the BSO-treated group compared to the placebo group whereas estradiol treatment caused an 8% increase in Ki67 staining (Figure 6c). Immunohistochemistry of paraffin-embedded tumor sections of mice treated with the combination of BSO and estradiol showed increased immunostaining for proteolytically cleaved PARP (marker for apoptosis) compared to control, estradiol, or BSO-treated groups (Figure 6d). Overall, these data show that BSO either alone or in combination with estradiol, reduces tumor growth by inhibiting proliferation and increasing apoptosis.

## Discussion

In the current study, we investigated whether suppression of the antioxidant glutathione by BSO has the ability to sensitize antihormone resistant MCF-7:2A breast cancer cells to estradiol-induced apoptosis. Our results showed that glutathione levels and the enzymes involved in its synthesis, glutathione synthetase and glutathione peroxidase, were significantly elevated in MCF-7:2A cells compared to parental MCF-7 cells and that suppression of glutathione by BSO sensitized these cells to estrogen-induced apoptosis *in vitro *and *in vivo*. The BSO-mediated estradiol-induced apoptosis was associated with a marked decrease in the expression of antiapoptotic Bcl-2 and Bcl-xL proteins and a significant increase in proapoptotic Bax protein. It is worth noting that high-dose estrogen was generally considered the endocrine therapy of choice for postmenopausal women with breast cancer prior to the introduction of tamoxifen, however, due to undesirable side effects, the use of high-dose estrogen was largely abandoned [29]. Here, we show that the killing effect of estradiol in antihormone resistant cells can be achieved at physiological concentrations when it is combined with non-toxic concentrations of BSO. Our present findings are consistent with previous studies which have shown that the cytotoxicity of a number of chemotherapeutic drugs, including melphalan [30], doxorubicin [31], and bleomycin [32], are significantly enhanced when glutathione is depleted by BSO.

An important target of BSO plus estradiol-induced apoptosis appears to be Bcl-2, whose protein expression was dramatically decreased in MCF-7:2A cells following glutathione depletion. Previous studies have shown that Bcl-2 functions as an antioxidant to block apoptosis and that Bcl-2 protein levels and glutathione intracellular concentration is coordinately regulated with a decrease in either favoring cell death [23,33]. It is believed that one mechanism by which Bcl-2 may function as an antioxidant is through upregulation of glutathione, leading to rapid detoxification of reactive oxygen species and inhibition of free radical-mediated mitochondrial damage. Bcl-2 also has the ability to shift the entire cellular redox potential to a more reduced state, which is independent of its effect on glutathione levels [33]. It is worth noting that glutathione levels and Bcl-2 protein expression were significantly elevated in MCF-7:2A cells compared to parental MCF-7 cells. In phase I trials [34,35], the concentration of BSO in blood has been shown to reach 0.5 to 1 mM, whereas, in mice [36,37] the concentration has been estimated to be 5 to 6 mM following an *in vivo *treatment of 4 mmol/kg. In our study, we showed that 100 μM BSO decreased glutathione concentrations by approximately 60% after 24 h and that BSO enhanced the apoptotic effect of estradiol in MCF-7:2A breast cancer cells as early as 48 h after treatment. Interestingly, treatment with BSO alone did not cause apoptosis in MCF-7:2A cells, indicating that glutathione depletion alone may not trigger apoptosis in these cells. This finding is consistent with previous studies by Mirkovic *et al*. [38] which showed that inhibition of glutathione by BSO did not increase susceptibility of mouse lymphoma cells to radiation-induced apoptosis even under conditions where glutathione levels were lowered by 50%. Other groups have made similar observations using BSO [39]. One possible explanation for this apparent contradiction might be the fact that BSO does not lower glutathione levels in mitochondria as effectively as it does in the cytoplasm [40]. Mitochondrial glutathione concentrations are regulated and have been implicated in apoptotic cell death [41], hence, it would be of interest to evaluate relative glutathione concentrations in the mitochondrial matrix of MCF-7:2A cells following treatment with BSO either alone or in combination with estradiol. Another possibility could be that cellular thiols other than glutathione may play important roles in regulating apoptosis [39]. The flavoprotein thioredoxin has been shown to be upregulated in several human tumors and is implicated in both cancer cell growth and apoptotic resistance [42]. However, it is not known whether Bcl-2 or other apoptotic regulators can influence the levels of thioredoxin or whether such modulation may contribute to resistance in human tumor cells.

Apart from Bcl-2, we also found that proapoptotic Bax protein was markedly increased in MCF-7:2A cells by the combination of BSO plus estradiol and this induction coincided with a loss of mitochondrial membrane integrity and cytochrome *c *release. Bax is normally found as a monomer in the cytosol of non-apoptotic cells and it oligomerizes and translocates to the outer mitochondrial membrane in response to apoptotic stimuli and induces mitochondrial membrane permeabilization and cytochrome *c *release [19]. In MCF-7:2A cells, Bax protein was induced as early as 24 h after BSO plus estradiol treatment (Figure 4) and suppression of Bax expression using siRNA was able to partially reverse the apoptotic effect of the combination treatment (data not shown). The induction of Bax coincided with cytochrome *c *release from the mitochondria into the cytosol, which was followed by activation of caspase 7, and PARP cleavage. It is worth noting that pretreatment of cells with the universal caspase inhibitor z-VAD almost completely blocked the apoptotic effect of BSO plus estradiol. It is also worth noting that antiapoptotic Bcl-2 and Bcl-xL proteins were also markedly decreased in MCF-7:2A cells following the combination treatment of BSO plus estradiol (Figure 4) and overexpression of Bcl-xL partially blocked the apoptotic effect of BSO plus estradiol (data not shown). This finding is important because there is evidence that suggests that the ratio rather than the amount of antiapoptotic vs proapoptotic proteins determines whether apoptosis will proceed [43]. Thus, it is reasonable to suggest that the apoptotic effect of BSO plus estradiol is mediated, in part, by the mitochondrial pathway through their ability to alter the ratio between proapoptotic and antiapoptotic proteins in target cells.

In addition to the mitochondrial pathway, BSO plus estradiol appears to induce apoptosis, in part, through activation of the JNK signaling pathway. JNKs are a group of mitogen-activated protein kinases (MAPKs) that bind the N-terminal activation domain of the transcription factor c-Jun and phosphorylate c-Jun on amino acid residues Ser63 and Ser73 [44]. JNKs are stimulated by multiple factors including cytokines, DNA-damaging agents, and environmental stresses and are important in controlling programmed cell death or apoptosis. The inhibition of JNKs has been shown to enhance chemotherapy-induced inhibition of tumor cell growth, suggesting that JNKs may provide a molecular target for the treatment of cancer [44]. We found that JNK activation (as measured by the increased levels of phospho-JNK1/2 and the JNK substrate phospho-c-Jun) correlated well with BSO plus estradiol-induced apoptosis in MCF-7:2A cells and pharmacologic disruption of this pathway using the JNK inhibitor SP600125 significantly attenuated this effect. Previously, Chen and coworkers [45] reported that BSO enhanced the apoptotic effect of arsenic (As_2_O_3_) in leukemia and lymphoma cells through activation of JNK and upregulation of death receptor (DR)5 and that inhibition of JNK by SP600125 decreased DR5 upregulation and apoptotic induction in U937 leukemia cells treated with arsenic plus BSO. While the exact mechanism by which JNK promotes apoptosis is not currently known, the phosphorylation of transcription factors such as c-Jun and p53, as well as pro- and antiapoptotic Bcl-2 family members [46] has been suggested to be of importance. It is worth noting that treatment with BSO plus estradiol markedly increased phosphorylated c-Jun in MCF-7:2A cells and decreased phosphorylated Bcl-2 in these cells. These findings thus suggest that BSO plus estradiol might mediate their apoptotic effect, in part, through activation of JNK.

## Conclusion

We have demonstrated that glutathione depletion by BSO sensitizes hormone-resistant MCF-7:2A human breast cancer cells to estradiol-induced apoptosis *in vitro *and *in vivo*. This finding has important clinical implications; particularly for the use of estrogen deprivation as long-term therapy, and it suggest that, if and when resistance develops, a strategy of treatment with estrogen combined with BSO may be effective in sensitizing resistant cells to apoptosis. It is worth noting that recently, Lonning and coworkers [9] reported a 33% complete response (that is, stable disease) with high dose diethylstilbestrol (DES) in postmenopausal patients with advanced breast cancer who were heavily pretreated with endocrine agents. However, 67% of the patients showed partial or no response [9] so the key to future clinical progress in the treatment of antihormone resistant breast cancer is to improve current treatment strategies. We are currently evaluating the optimal dose of daily estradiol therapy to reverse antihormonal resistance [4] but the goal is to enhance the estradiol-induced apoptotic response. The present findings suggest that BSO is indeed capable of enhancing the apoptotic effect of estradiol in antihormone resistant breast cancer cells. It is worth noting that a phase I study of BSO administered with the anticancer drug melphalan showed that continuous-infusion of BSO was relatively nontoxic and resulted in depletion of tumor glutathione [35,47]. Thus it is possible that future clinical studies of BSO infusions combined with low dose estrogen hold the promise of improving disease control for patients with antihormone resistant ER-positive metastatic breast cancer.

## Abbreviations

BSO: L-buthionine sulfoximine; E_2_: 17β-estradiol; ER: estrogen receptor; FBS: fetal bovine serum; GCS: glutamylcysteine; GPx2: glutathione peroxidase; GS: glutathione synthetase; GSH: glutathione; H&E: hematoxylin and eosin; JNK: c-Jun N-terminal kinase; Rh123: rhodamine 123; SFS: dextran coated charcoal-treated FBS; TUNEL: terminal deoxynucleotidyl transferase-mediated dUTP nick end-labeling.

## Competing interests

The authors declare that they have no competing interests. The views and opinions of the author(s) do not reflect those of the US Army or the Department of Defense.

## Authors' contributions

JSLW designed and coordinated the studies, analyzed the data and interpreted the results, generated the figures, and wrote and revised the manuscript. HK performed the cell proliferation assays and the western blots. CW performed the glutathione assay. RP and JP performed the animal experiments. AJK performed the immunohistochemistry. VCJ is the Principal Investigator (PI) of the laboratory in which all experiments were conducted and is the recipient of the grant that partially funded the project. VCJ was instrumental in revising the manuscript. All authors read, assisted in revision and approved the final manuscript.

## Supplementary Material

Additional file 1 Powerpoint file showing the growth inhibitory effect of buthionine sulfoximine (BSO) and 17β-estradiol (E_2_) in MCF-7:2A cells is reversed by the antiestrogen 4-hydroxytamoxifen (4-OHT). MCF-7:2A cells (30,000/well) were seeded in 24-well plates and after 24 h were treated with < 0.1% ethanol vehicle (control), 1 nM E_2_, 100 μM BSO, 100 μM BSO plus 1 nM E_2_, 1 μM 4-OHT, 4-OHT + E_2_, 4-OHT + BSO, 4-OHT + E_2 _+ BSO for 7 days. At the indicated time points, cells were harvested and total DNA (μg/well) was quantitated as described in Materials and methods. The data represent the mean of three independent experiments; bars, ± standard error of the mean (SEM). *, p < 0.01 compared with control cells; #, p < 0.01 compared to E_2_-treated cells. Click here for file
