# Correction to: Buthionine sulfoximine sensitizes antihormone-resistant human breast cancer cells to estrogen-induced apoptosis

**DOI:** 10.1186/s13058-018-0986-y

**Published:** 2018-06-14

**Authors:** Joan S. Lewis-Wambi, Helen R. Kim, Chris Wambi, Roshani Patel, Jennifer R. Pyle, Andres J. Klein-Szanto, V. Craig Jordan

**Affiliations:** 1 Department of Medical Sciences, Fox Chase Cancer Center, 333 Cottman Avenue, Philadelphia, PA 19111 USA; 2 Department of Surgical Oncology, Fox Chase Cancer Center, Philadelphia, USA; 3 Department of Radiation Oncology, University of Pennsylvania, Philadelphia, USA; 4 Department of Pathology, Fox Chase Cancer Center, Philadelphia, USA

## Correction

After the publication of this work [1] an error was noticed in Fig. 3a and Fig. 5a. In Fig. 3a, the TUNEL staining image for the E2-treated MCF-7:2A cells was accidentally duplicated for the image for the BSO-treated MCF-7:2A cells. We have repeated this experiment using the Click-it-TUNEL kit under the same conditions previously described in our original publication [1] and our results are consistent. Our revised Fig. 3a showed that BSO treatment significantly enhanced E2-induced apoptosis in anti-hormone-resistant MCF-7:2A breast cancer cells compared to E2 or BSO treatment alone, however, in wild-type MCF-7 cells BSO treatment did not significantly alter the growth of these cells either alone or in combination with E2. The corrected Fig. 3a is shown below. Similarly, we also noticed an error in the Western blot shown in Fig. 5a. Specifically, there was a duplication of the phospho-JNK blot for the MCF-7 cells and MCF-7:2A cells for the control, E2, and BSO-treated groups (top blot). To correct this error, we repeated this experiment using the same conditions described in our original publication [1] and the revised Fig. 5a is shown below. We found that BSO combined with E2 dramatically increased phospho-JNK, phospho-c-Jun, and c-Jun expression in MCF-7:2A cells but not in wild-type MCF-7 cells which is consistent with our previous findings in our original publication [1]. Our revisions validate our previous findings and are consistent with the conclusions stated in our original publication. We apologize for these two errors.

**Fig. 3:**
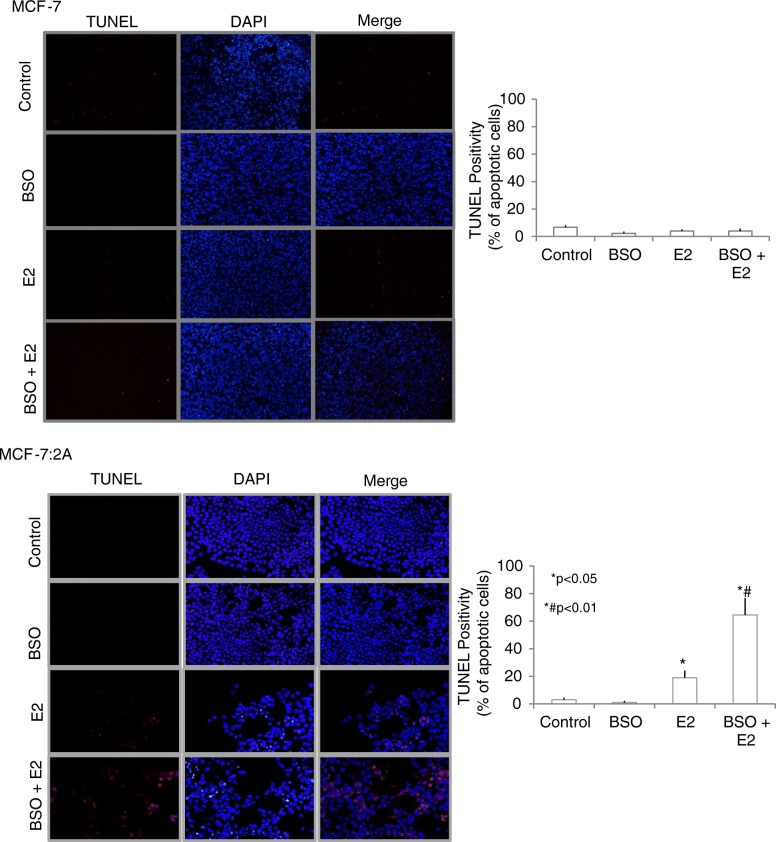
Buthionine sulfoximine (BSO) plus estradiol induce apoptosis in MCF-7:2A cells. **a** Terminal deoxynucleotidyl transferase-mediated dUTP nick end-labeling (TUNEL) staining was used to detect apoptosis in MCF-7 (top panel) and MCF-7:2A cells (bottom panel) following BSO plus 17β-estradiol (E_2_) treatment for 96 h. The Click-iT™ Plus TUNEL kit (Thermo Fisher Scientific) was used instead of the *TUNEL* In Situ Cell Death kit conjugated with horse-radish peroxidase (POD) (Roche Applied Science, Indianapolis, IN, USA) as previously described in the original publication [1]. TUNEL-positive cells were stained red and the nuclei were counterstained with DAPI (blue). Merged images are shown at right with overlapping DAPI in blue and Alexa Fluor 594 in red. Slides were photographed through a fluorescence microscope under 20× magnification. Average intensity of TUNEL signal was taken from 3 images quantified using red color channel on Image J. Bar graphs (right) quantify apoptosis (TUNEL-positive cells), which is expressed as percentage of apoptotic cells for each condition. Data are presented as mean ± standard error (*n* = 3). Mean ± SEM); *, *p* < 0.001 significantly different from control cells; ^#^, *p* < 0.01 significantly different from E_2_ or BSO alone

**Fig. 5:**
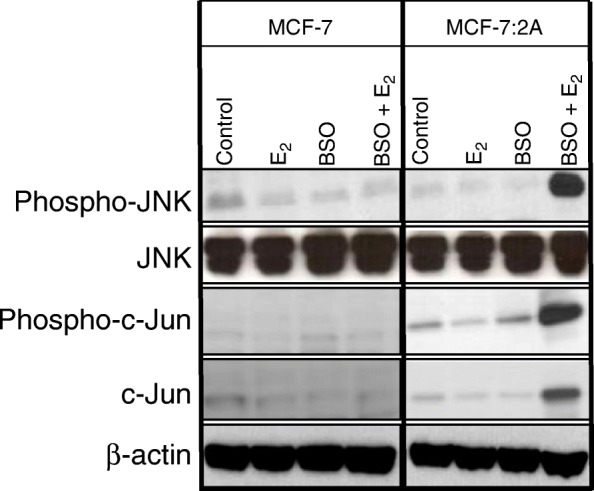
Activation of c-Jun N-terminal kinase (JNK) signaling pathway in MCF-7 and MCF-7:2A cells in response to buthionine sulfoximine (BSO) and 17β-estradiol (E_2_) treatment. **a** MCF-7 and MCF-7:2A cells were treated with ethanol vehicle (control), 1 nM E_2_, 100 μM BSO, or BSO plus E_2_ for 48 h and protein levels of phosphorylated JNK, JNK, phosphorylated c-Jun, and c-Jun were analyzed by western blotting. β-Actin was used as a loading control. Data shown is representative of 3 separate experiments yielding similar results
