# Supplementary material for: Buthionine sulfoximine sensitizes antihormone-resistant human breast cancer cells to estrogen-induced apoptosis
Source: Breast Cancer Res. 2008 Dec 5;10(6):R104. doi: 10.1186/bcr2208 (PMC2656901; doi:10.1186/bcr2208)
Supplement: Additional file 1 — Powerpoint file showing the growth inhibitory effect of buthionine sulfoximine (BSO) and 17β-estradiol (E2) in MCF-7:2A cells is reversed by the antiestrogen 4-hydroxytamoxifen (4-OHT). MCF-7:2A cells (30,000/well) were seeded in 24-well plates and after 24 h were treated with < 0.1% ethanol vehicle (control), 1 nM E2, 100 μM BSO, 100 μM BSO plus 1 nM E2, 1 μM 4-OHT, 4-OHT + E2, 4-OHT + BSO, 4-OHT + E2 + BSO for 7 days. At the indicated time points, cells were harvested and total DNA (μg/well) was quantitated as described in Materials and methods. The data represent the mean of three independent experiments; bars, ± standard error of the mean (SEM). *, p < 0.01 compared with control cells; #, p < 0.01 compared to E2-treated cells. [file bcr2208-S1.ppt]

## Slide 1
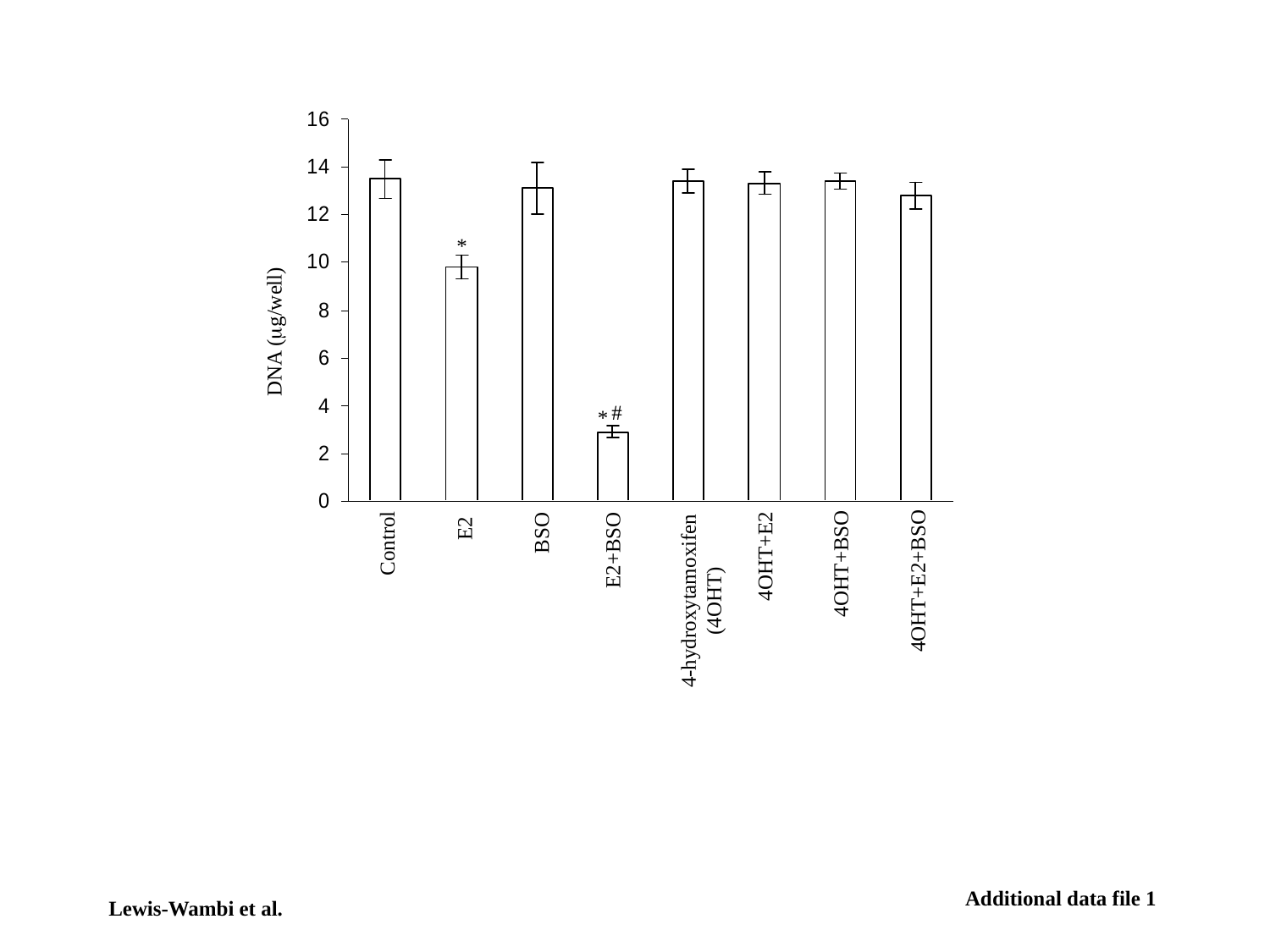

*
DNA (μg/well)
#
*
BSO
Control
 E2
E2+BSO
4OHT+E2
4OHT+BSO
4OHT+E2+BSO
4-hydroxytamoxifen
(4OHT)
Additional data file 1
Lewis-Wambi et al.
